# Patient needs and preferences for herb-drug-disease interaction alerts: a structured interview study

**DOI:** 10.1186/s12906-017-1630-6

**Published:** 2017-05-19

**Authors:** Carrie M. Christensen, Rebecca S. Morris, Seraphine Chepkemoi Kapsandoy, Melissa Archer, Jinqiu Kuang, Laura Shane-McWhorter, Bruce E. Bray, Qing Zeng-Treitler

**Affiliations:** 1 Department of Biomedical Informatics, University of Utah School of Medicine, 421 Wakara Way, Suite 140, Salt Lake City, UT 84108 USA; 2 Drug Regimen and Review Center, University of Utah College of Pharmacy, Salt Lake City, UT USA; 3 Department of Pharmacotherapy, University of Utah College of Pharmacy, Salt Lake City, UT USA

**Keywords:** Alerts, Complementary and alternative medicine, Structured interview study

## Abstract

**Background:**

While complementary and alternative medicine (CAM) is commonly used in the United States and elsewhere, and hazardous interactions with prescription drugs can occur, patients do not regularly communicate with physicians about their CAM use. The objective of this study was to discover patient information needs and preferences for herb-drug-disease interaction alerts.

**Methods:**

We recruited 50 people from several locations within the University of Utah Hospital to participate in this structured interview study. They were asked to provide their preferences for the herb-drug-disease interaction alerts. Qualitative methods were used to reveal the themes that emerged from the interviews.

**Results:**

Most participants reported they had previously used, or they were currently using, CAM therapies. The majority had made the effort to inform their healthcare provider(s) about their CAM usage, although some had not. We found that most respondents were interested in receiving alerts and information about potential interactions. Many preferred to receive the alerts in a variety of ways, both in person and electronically.

**Conclusions:**

In addition to conventional medicine, many patients regularly use complementary and alternative therapies. And yet, communication between patients and providers about CAM use is not consistent. There is a demand for interventions in health care that provide timely, integrative communication support. Delivering the herb-drug-disease alerts through multiple channels could help meet critical patient information needs.

**Electronic supplementary material:**

The online version of this article (doi:10.1186/s12906-017-1630-6) contains supplementary material, which is available to authorized users.

## Background

According to the National Center for Complementary and Integrative Health (NCCIH), “Many Americans—more than 30% of adults and about 12% of children-use health care approaches outside of mainstream Western, or conventional, medicine.” “Complementary” medicine uses methods that are outside of the mainstream, together with conventional medicine. When an approach is used in place of conventional medicine it is called “alternative.” [1].

CAM use may include dietary supplements or other products taken by mouth. These dietary supplements contain ingredients (such as vitamins, minerals, herbs or botanicals, amino acids, concentrates, metabolites, constituents, or extracts) that are intended to supplement an individual’s diet [2]. Some supplements ensure you will get an adequate amount of essential minerals and nutrients in your diet, for instance, while others may contain properties that reduce your disease risk. They may be used together with, or in place of, conventional medications.

The use of CAM is widespread among relatively healthy people, as well as among those who suffer from disease. A survey of 20,000 individuals indicated that supplements are used by 49% of the United States population (53% of females and 44% of males) [3]. Approximately $15 billion dollars is spent per year on dietary supplements [4]. In the United States, approximately 4 out of every 10 adults, and 1 out of 9 children, use some form of CAM [5]. CAM use is most prevalent in women, and individuals with higher incomes and higher levels of education [5].

The NCCIH currently sorts most complementary health approaches into one of two subgroups, “natural products” or “mind and body practices.” A survey from 2007 reported that U.S. adults commonly used nonvitamin, nonmineral (NVNM) dietary supplements (17.7%), such as echinacea, St.John’s wort, or ginseng, and various mind body practices such as deep breathing exercises (12.7%), meditation (9.4%), chiropractic or osteopathic manipulation (8.6%), massage (8.3%), and yoga (6.1%) [5].

Many CAM products and practices are believed to be safe for consumers, although “natural products” can interact with prescription medications to negatively impact health outcomes for patients [6]. There are many known interactions between supplements and conventional medications which present health risks [7]. For instance, ginseng is known to interact with warfarin and may increase risk of breakthrough thromboembolic events [7]. St John’s wort may interact with certain medications (antihypertensives, statins, oral contraceptives, and anticoagulents like warfarin) lowering serum concentrations to sub-therapeutic levels, and thus diminishing the benefits conferred by those medications [7].

It has been estimated that only 33.4% of the general public informs health care providers about their use of supplements [8]. Our previous study showed that patient-reported CAM usage rates and provider estimates are disparate [9]. A concern is that many individuals are taking products that are contraindicative or seriously endangering to patients with conditions such as cardiovascular disease [10] and diabetes [11].

This paper describes a structured interview study that was conducted to gain insight into preferences for an alert system we are developing that will inform patients and physicians of imminent herb-drug-disease interactions. Alerts and reminders have been variably successful informatics interventions in healthcare [12–15], though drug-drug interaction alerts have generally been directed at providers rather than patients. Herb-drug-disease alerts are a fairly new domain for research and development. We need to understand the patient-centered information needs and preferences of the target population in order to design effective alerts.

## Methods

We recruited a convenience sample of accessible participants from several University of Utah locations until we felt the sample group included a fair representation of underserved minorities and a sufficient number was reached to reveal patterns and themes associated with preferences for CAM interaction alerts. Qualitative methods were used to reveal the themes which emerged from the interviews, and we used qualitive methods to interpret and integrate the findings of this interview study.

The study was pre-approved by the University of Utah Institutional Review Board (IRB). We included participants age 21 or older, with the ability to speak and read English. Members of the University of Utah Health Care System such as physicians, physician assistants, nurse practitioners, students, and administrative staff were excluded from the study.

The research team explained the purpose of the interview to those interested in participating in the study. A short introduction was also provided explaining the prevalence of CAM use and the potential for CAM treatments to interact with conventional medications and serious health conditions. Those who agreed to take part in the study read the consent form and gave their consent by verbal agreement. Participants then filled out a demographic questionnaire. Names or other personal identifiers were not recorded.

During the interviews, research staff members asked a series of questions according to the study script (see Additional file 1). The questions were either multiple choice or short answer format. The answers from study participants were recorded in handwritting by the staff. In order to ensure that all interviews were conducted in a standardized format, a staff training session was held before patient recruitment.

Notes from the interviews were transcribed into an electronic datasheet for analysis. Descriptive statistics were generated for the demographic information and participant responses to the multiple choice questions. Short answer responses were coded to refine categories, themes, and concepts. Two researchers worked to complete the qualitatitive analysis. The qualitatitive transcripts were individually and jointly analyzed to develop a coding manual of themes and definitions. The qualitative data was processed using content analysis techniques and Atlas ti software, version 7.0. Their work was checked for interrater reliability and validity.

## Results

In this convenience sample of 50 respondants (see Additional file 2), the demographic data showed that there were more white (66%) and female (64%) participants than there were non-white (34%) and male (36%). Most of the participants (80%) had more than a 12th grade education and (88%) spoke English as their first language. The majority of respondents were 30 to 59 years of age. Compared to the general U.S. population, our study sample had achieved relatively higher levels of education, yet they were similar in racial diversity [16]. Table 1 shows the demographic data collected from the study participants.

**Table 1:** Participant demographics by CAM use

	Total (*N* = 50)	CAM users (*N* = 43)	Non-CAM users (*N* = 7)
Sex- no. (%)			
Men	18 (36)	16 (37.21)	2 (28.57)
Women	32 (64)	27 (62.79)	5 (71.43)
Age- no. (%)			
21–29 years old	6 (12)	5 (11.63)	1 (14.29)
30–39 years old	11 (22)	10 (23.26)	1 (14.29)
40–49 years old	8 (16)	8 (18.60)	0 (0)
50–59 years old	12 (24)	10 (23.26)	2 (28.47)
60–69 years old	7 (14)	5 (11.63)	2 (28.57)
70–79 years old	4 (8)	3 (6.98)	1 (14.29)
80+ years old	1 (2)	1 (2.33)	0 (0)
Unknown	1 (2)	1 (2.33)	0 (0)
Ethnicity- no. (%)			
Hispanic	7 (14)	7 (16.28)	0 (0)
Non-Hispanic	43 (86)	36 (83.72)	7 (100)
Race- no. (%)			
White	33 (66)	27 (62.79)	6 (85.71)
Black	7 (14)	7 (16.28)	0 (0)
Other	10 (20)	9 (20.93)	1 (14.29)
Education- no. (%)			
12^th^ Grade	10 (20)	7 (16.28)	3 (42.86)
Above 12^th^ Grade	40 (80)	36 (83.72)	4 (57.14)
Language- no. (%)			
English	44 (88)	37 (86.05)	7 (100)
Non-English	6 (12)	6 (13.95)	0 (0)

We found many subjects (86%) had previously used or currently use complementary or alternative medicine. This prevalence is higher than what was reported by a national survey [5], yet consistent with our previous study in Utah [11]. Table 2 displays the most commonly used supplements or alternative medicine therapies. Table 3 allows for the comparison between the total number of CAM therapies or supplements reported by all 50 respondents and use of multivitamins and other vitamin or mineral supplements.

**Table 2:** Supplement or alternative medicine therapy and frequency of use (*N* = 43)

Percent use	
72.1%	Vitamin or mineral supplements other than multivitamins (Vitamin C, Niacin, Vitamin D, etc.)
53.5%	Multivitamin
23.3%	Fish oil or omega-3
14.0%	Probiotics
14.0%	Over-the-counter medications (Aspirin, Tylenol, etc.)
11.6%	Glucosamine or chondroitin sulfate
9.3%	Tea
7.0%	CoQ10
7.0%	Flax seed
60.5%	Other supplements or alternative medicine therapies (wellness formula from health food store, vegi protein powder, laxative, etc.)

**Table 3:** Number of CAM therapies or supplements by multivitamin and other vitamin or mineral supplement use

Total no. of CAM therapies or supplements	Any CAM use	Uses vitamin or mineral supplements (other than multivitamins)		Uses multivitamins	
		Yes	No	Yes	No
0	7 (14.0)	0 (0.0)	7 (14.0)	0 (0.0)	7 (14.0)
1	7 (14.0)	2 (4.0)	5 (10.0)	3 (6.0)	4 (8.0)
2–3	12 (24.0)	5 (10.0)	7 (14.0)	6 (12.0)	6 (12.0)
4–5	9 (18.0)	9 (18.0)	0 (0.0)	4 (8.0)	5 (10.0)
≥6	15 (30.0)	15 (30.0)	0 (0.0)	10 (20.0)	5 (10.0)
Total	50 (100)	31 (62.0)	19 (38.0)	23 (46.0)	27 (54.0)

### Communication with medical providers about CAM use

A majority of the respondents who had used complementary or alternative medicine and some also told their doctor or other health care provider(s) about their CAM use. Being asked for the information by health care professionals (40.7%) and caution/safety concerns (25.9%) were the two main reasons the participants gave for having told about their CAM use. Seven themes were identified in all:

1. Health care professional requested the information:
  RESPONDENT 24. Because he asked. I don’t want anything to interact. I always tell.
2. Caution/safety concerns:
  RESPONDENT 25. I have a list of everything I take because it could interact with my prescription.
3. Knowing that it was important information:
  RESPONDENT 34. Because I am a medical professional and I know it’s important.
4. Desire to consult with a physician on current medical problem(s):
  RESPONDENT 39. Yes, because she [doctor] could see the change in me and I wanted to share. Everything turned around. I was going to do chemotherapy and have lymph nodes removed. The body has its own consciousness and healing.
5. Mistrust of medical doctors or feeling that the information is not relevant:
  RESPONDENT 36. Sometimes, but I do not list it on my forms of medications. I tell them, depending on how I feel, if it is pertinent to my treatment. For example, if I am talking about having cramps I tell them about my digestive enzymes.
6. Health concerns related to comorbidities and/or medications:
  RESPONDENT 35. I make them aware because I have prescription medicines.
7. Influence of a family member:
  RESPONDENT 37. My wife told me to. I never would have thought about telling them before.

Potential interactions are most likely to occur when patients start or stop taking supplements or alternative medicine. These 27 participants informed their providers of their CAM usage at various times as shown in Table 4. The most common occasion during which this information was conveyed was when they filled out a medical history form at a doctor appointment (85.2%).

**Table 4:** When participants informed provider of CAM use (*N* = 27)

	Frequency
When they started taking them	29.6%
When they stopped taking them	7.4%
When they suspected a problem	11.1%
When they filled out their history at the doctor’s visit	85.2%
Other (examples include: randomly, at health care provider visit, when concerned about potential overdose, when filling prescriptions, and when they wanted to be on supplements)	37.0%

### Barriers to communication with providers about CAM use

Of the 43 respondents who had used complementary or alternative medicine, 16 did not inform their doctor or other health care provider(s). Three major reasons were identified:

1. Never asked about their use of supplements or alternative medicine:
  RESPONDENT 21. They have never asked. I didn’t think it was a big deal.
2. Perceived unimportance or irrelevance
  RESPONDENT 44. I guess I just never saw it as important.
3. Partially informed health care provider of supplement use:
  RESPONDENT 41. Yes, she (doctor) is aware of the vitamin D. Hasn’t told her about vitamin B yet.

Half of the participants did not tell their doctor because they viewed the information as unimportant or irrelevant. People are more likely to tell their providers if they are asked for the information, yet only 52% of the study population had ever been asked. If CAM information is inconsistently collected by providers. Study participants who had been asked by a health care professional about their CAM use in the past were asked to give examples of when this occurred. Many respondents said that health care professionals had asked them verbally or through questionnaires at intake. Others were asked when discussing medication history, or upon being prescribed a new medication. Some told their physicians during a clinical consult regarding a possibly-related health concern.

If asked about their supplement or alternative medicine use, 49 out of 50 participants said that they would tell their providers. This reveals a strong willingness on the part of consumers to share their CAM usage information with providers. However, the participants were not completely confident that their providers would use the information to enhance their medical care. Only half of the respondents felt that their providers would take their CAM usage into account when treating or managing their illness. The alerts we provide could be useful for conveying critical information, hopefully without overwhelming the patient.

### Needs and preferences for interaction alerts

Most of the interview respondents said that they were interested in receiving information about potential interactions with their prescriptions and other medicines, including herbal supplements. Their needs and preferences were categorized into the following groups:

1. Need for general information about ingredients, effects and interactions:
  RESPONDENT 4. The more information the better. If you don’t know about the potentials you could be increasing/decreasing the potency and interactions.
2. Caution/safety concerns:
  RESPONDENT 1. I don’t want to get sicker than I already am. A lot can interact and kill you, alternative and otherwise.
3. Mistrust of healthcare provider(s):
  RESPONDENT 37. I would like to know that even if it’s missed by the doctor or pharmacy I would still get the information and bring it to someone’s attention; fail-safe type thing.
4. Financial concerns:
  RESPONDENT 28. Well, what if I find out it’s just a waste of money [and] cancelling out the effect of another medicine.
5. Desire to consult with a physician:
  RESPONDENT 39. Yes, I would want to know what the doctor thought, to get another perspective. [She] doesn’t want to limit self. Has seen miracles happen.
  46 out of 50 interview participants were interested in the information.

The most common themes were general information needs and caution/safety concerns. Acceptable methods by which participants wished to receive information alerting them to potential interactions are listed in Table 5. The participants were asked if they wanted to be told about all possible interactions, or only the most serious. 39 participants (78%) chose to be told about all possible drug interactions. The remaining 11 wanted to be informed about serious drug interactions only.

**Table 5:** Participant answers to multiple choice question about preferred methods to receive interaction alerts (*N* = 50)

	Frequency
Directly from their doctor	76%
At a kiosk in the waiting room at doctor visits	44%
Through a smart phone application	58%
Through their personal health record (PHR)	64%
At the pharmacy	74%
By print or email	76%
Other (answers included: written, verbal, phone, face-to-face, website, TV in waiting room, software program for PC, during annual physical exam, email only, and at home)	28%

The participants considered life-threatening interactions to be serious, as well as those causing permanent organ damage. Vision or blood pressure changes, urinary retention, allergic reactions, and interactions causing problems with prescriptions and other drugs were also considered serious. The preferences of those who wanted to know about all possible drug interactions have been categorized into the following 5 groups:

**Table 6:** Patient preferences regarding types of information they would like to receive (*N* = 50)

	Frequency
Taking St. John’s wort with warfarin frequently results in an interaction	82%
Taking St. John’s wort with warfarin can cause a serious reaction.	88%
We strongly recommend that you inform your doctor and consider not taking St. John’s wort	90%
You are at greater risk because you have high blood pressure and have experienced an episode of bleeding	94%
Taking St. John’s wort with warfarin can cause you to have a stroke	96%
Taking St. John’s wort with warfarin decreases the clotting ability of your blood	88%
A recent article described two cases where patients who were taking St. John’s wort with warfarin had strokes	62%

1. To be well informed:
  RESPONDENT 18. You want all information, every detail. If you don’t get it all you get poor information.
2. Awareness of possible interactions and their severity levels:
  RESPONDENT 33. When you say that I see a scale, e.g., yellow, green, red. I want to see a scale/grading scale of severity so I can get an idea of what I am dealing with.
3. Decision support:
  RESPONDENT 35. Because I want to know about my health so I can make a good decision.
4. To avoid interactions:
  RESPONDENT 4. Concerned about interactions that lessen or increase the potency of the given dosage. That is, if I’m on X mg of an antidepressant, maybe the herbal medication will decrease the effectiveness. […].
5. Mistrust of clinicians:
  RESPONDENT 14. [He] plans ahead for different disease contingencies. Looks ahead, feels doctors use patients as guinea pigs. […]. If you’re considered a burden to the system, they don’t care. [He] stays away from herbal medications because doesn’t know what they’ll do to him personally.

Of these five themes, the largest group of respondents preferred to know about all possible interactions in order to be well informed (category 1), followed by those who wanted the information for decision support (category 3), and those who wanted to be aware in order to consider the severity levels (category 2). The data listed in Table 6 displays the respondents’ preferences upon being asked, “What information would you like to receive regarding the possible drug interaction?” Over half wanted to receive all available information. One person indicated they did not wish to receive any information about possible interactions. The interview participants were asked to describe the reasons why they selected to receive the information they did. Their responses were categorized as follows:

1. Safety:
  RESPONDENT 15. […]. I don’t want to have a stroke. I would be really interested. Need to know all possible after-effects. […]. I am chemically sensitive.
2. Increased knowledge:
  RESPONDENT 11. To get knowledge about the medications, to see if it made [them] stronger.
3. To be proactive:
  RESPONDENT 31. Being proactive about our health.
4. Consult with primary care provider:
  RESPONDENT 8. Because it would help to know when you were going to the doctor, so they wouldn’t give you something you would have a bad reaction to.
5. Mistrust of clinicians, pharmacy, drugs not approved by the FDA:
  RESPONDENT 40. Doctors should not act like God or something. They should listen to patients and take their advice on what works for their bodies. […].
6. To avoid death:
  RESPONDENT 17. Because I don’t want to die.
7. Personalization:
  RESPONDENT 15. [I] especially like to be told things special for me. […].

The interviews we conducted gathered information on patient needs and preferences concerning CAM interaction alerts. There was a marked desire for information on the part of consumers. Almost all participants (92%) expressed an interest in receiving information on interaction alerts and most (78%) wanted to be told about all possible drug interactions (in addition to learning about the most serious interactions). Interview participants wished to receive alerts in a variety of ways, in-person and electronically.

## Discussion

### Significance

The structured interview study we conducted elicited patients’ needs and preferences for CAM herb-drug-disease interaction alerts. We observed strong consumer demand for the interaction alerts. Most expressed an interest in receiving information on potential interactions, and most wanted to be told about all possible drug interactions, as opposed to only the most serious interactions. Similarly, over half of the respondents elected to receive all of the seven types of information we presented regarding possible drug reactions. Participants also preferred to receive the interaction alerts in a variety of ways, electronically as well as in personally. The alert system we envision is inclined toward redundancy. Patients should get information from their doctors directly, as well as from the alert messages they receive through a convenient device such as their smart phone.

Several previous studies have investigated CAM useage in different patient populations and patient/physician communication about CAM use. We found that supplements were used commonly by interview participants, and there is a deficiency in communication between patients and providers about it. These findings are consistent with the prior research. Patient perspectives on herb-drug-disease interaction alerts have not been investigated specifically. Our research group did conduct a systematic literature review on alerts and reminders targeting patients (Perri-Moore S [17]). While alerts are still rare in the literature, patient reminders are generally received with positive results.

### Limitations

This moderately sized qualitative study elicited the interests of participants from a patient-centered perspective. We asked the interview participants about hypothetical alert situations; their preferences for interaction alerts in this study may not be completely true to real-life circumstances. For instance, a small number of patients might receive excessive alerts and experience alert fatigue (like physicians). Another concern is that directly alerting patients might cause them to act in ways that are dangerous to their health; they could elect to stop taking their prescription drug, for instance, instead of the problematic supplement. In this interview study we did not explore the actions taken following alerts. It is recommended that the interaction alerts we propose always remind patients to consult with their physicians.

### Future work

The findings of this study will inform our ongoing project to alert patients of potentially harmful interactions. In the near future we will explore the actions taken by patients (and providers) following the herb-drug-disease interaction alerts. We will design our system to meet patient needs, such as providing comprehensive information through a variety of communication channels.

Consistent with prior literature on the subject, we observed a high prevalence of CAM use, and communication between patients and providers on the subject was inconsistent. This study highlights the need to develop informative interventions that will hold interest and protect patients by preventing harmful interactions. The study findings indicate that patients are willing to partner with physicians regarding decision-making about CAM use, though physicians are not fully trusted to always take CAM usage into account when creating a treatment plan. Providing alerts to inform patients of potential herb-drug-disease interactions, and delivering them through several methods, would help meet patient information needs.

## Conclusions

Consistent with prior literature on the subject we observed a high prevalence of CAM usage in the interview study population, and communication with providers about such use was unreliable. The study findings indicated that patients would be willing to partner with their physicians in decision-making about CAM, although physicians are not fully trusted to take CAM useage into consideration. As we move toward integrative health, there is a need in the community to develop interventions, alerts, which will inform patients and warn them of possible conflicts with their prescriptions and their complementary and alternative medicines.

## Additional files

Additional file 1: Participant Data Collection Sheet and Interview Script. (PDF 165 kb)

Additional file 2: Patient Questionnaire. (XLSX 31 kb)

## Abbreviations

CAM: Complementary and Alternative Medicine
IRB: Institutional Review Board
NCCIH: National Center for Complementary and Integrative Health
NVNM: Nonvitamin, Nonmineral
