# Supplementary material for: Patient needs and preferences for herb-drug-disease interaction alerts: a structured interview study
Source: BMC Complement Altern Med. 2017 May 19;17:272. doi: 10.1186/s12906-017-1630-6 (PMC5438528; doi:10.1186/s12906-017-1630-6)
Supplement: Supplementary file 1 — Participant Data Collection Sheet and Interview Script. (PDF 165 kb) [file 12906_2017_1630_MOESM1_ESM.pdf]

Participant Data Collection Sheet

Demographic Information

Participant ID ___________________

Gender:
- ☐ Male
- ☐ Female

Age bracket:
- ☐ 21-29 years old
- ☐ 30-39 years old
- ☐ 40-49 years old
- ☐ 50-59 years old
- ☐ 60-69 years old
- ☐ 70-79 years old
- ☐ 80 years old or older

Ethnicity:
- ☐ Hispanic
- ☐ Non-Hispanic

Race:
- ☐ American Indian/Alaska Native
- ☐ Asian
- ☐ Native Hawaiian or Other Pacific Islander
- ☐ Black or African American
- ☐ White
- ☐ Other

Education:
- ☐ 4th Grade
- ☐ 8th Grade
- ☐ 12th Grade
- ☐ Above 12th Grade

First Language: ____

Interview Questions

1. Have you ever used or do you currently use supplements or alternative medicine, such as: vitamins, minerals, herbs, plants, or other natural products, amino acids or over-the-counter medication?

    a. Yes if so, what?

    b. No

2. Have you ever told your doctor or health care provider about your use of, supplements or alternative medicine?

    a. Yes, why?
    ____
    ____
    ____
    ____
    ____
    ____

    b. No, why not?
    ____
    ____
    ____
    ____
    ____
    ____

3. If yes, when did you tell your doctor or health care provider about your supplements or alternative medicine use? For example (circle all that apply)
    a. When you started taking them
    b. When you stopped taking them
    c. When you suspected a problem
    d. When you filled out your history at the doctor's visit
    e. Other:______________________________
    ____
    ____
    ____
    ____

4. Have any doctors or other health care providers ever asked you about your use of supplements or alternative medicine? If yes, please provide an example of when this occurred.

a. Yes, please provide an example of when this occurred.

b. No

5. If you are asked about your supplements or alternative medicine use, do you tell your doctor or health care provider?

6. If you do inform your doctor or health care provider, do you feel the information is taken into account with regard to your treatment or management of your illness?

7. Would you be interested in receiving information about potential interactions with your prescriptions and other medications, including supplements and alternative medicine?

    a. Yes, why?

    ____
    ____
    ____
    ____
    ____

    b. No, why not?

    ____
    ____
    ____
    ____
    ____

8. If we send you information alerting you about potential interactions, when and how would you like to receive this information? For example, would you prefer to receive information (circle all that apply)

    a. Directly from your doctor
    b. At a kiosk in the waiting room at doctor visits

c. With a smart phone application
d. Through your personal health record (PHR)
e. At the pharmacy
f. By print or email
g. Other, please explain

9. Some interactions are severe, while others are less severe. Do you want to be told about **ALL** possible interactions, or just the more serious interactions? Some interactions may be life threatening, for example, while other interactions may be less severe though occurring with greater frequency. Please select a. or b. below and tell us about your preferences:

    a. **ALL** possible interactions. Please tell us why you would like to know about all possible interactions.

    b. **SERIOUS** interactions only. Please tell us which types of interactions you would like to be told about specifically.

10. What information would you like to receive regarding the possible drug interaction? For example, if you were taking St. John’s wort with warfarin (a blood thinner) which of the information below would you like to receive? (circle all that apply)

    a. Taking St. John’s wort with warfarin frequently results in an interaction.
    b. Taking St. John's wort with warfarin can cause a serious reaction.
    c. We strongly recommend that you inform your doctor and consider not taking St. John's wort.
    d. You are at greater risk because you have high blood pressure and have experienced an episode of bleeding.
    e. Taking St. John's wort with warfarin can cause you to have a stroke.
    f. Taking St. John's wort with warfarin decreases the clotting ability of your blood.
    g. A recent article described two cases where patients who were taking St. John's wort with warfarin had strokes.

11. Please describe your reasons for selecting your responses to the previous question.
